# DETERMINANTS OF PHYSICAL ACTIVITY IN COMMUNITY-DWELLING OLDER ADULTS: AN UMBRELLA REVIEW

**DOI:** 10.1093/geroni/igad104.2307

**Published:** 2023-12-21

**Authors:** Cassandra D’Amore, Stephanie Saunders, Neera Bhatnagar, Lauren Griffith, Julie Richardson, Marla Beauchamp

**Affiliations:** McMaster University, Hamilton, Ontario, Canada; McMaster University, Hamilton, Ontario, Canada; McMaster University, Hamilton, Ontario, Canada; McMaster University, Hamilton, Ontario, Canada; McMaster University, Hamilton, Ontario, Canada; McMaster University, Hamilton, Ontario, Canada

## Abstract

Physical activity (PA) is an important modifiable factor for noncommunicable diseases, mental health, and functional trajectories as we age. The World Health Organization highlights the need to mobilize policies targeted toward modifiable determinants of healthy aging. Therefore, understanding the current evidence for PA determinants is crucial to achieving these goals. This umbrella review aimed to summarize the determinants of PA in older adults. A research librarian searched six databases. Systematic and scoping reviews were included if they investigated community-dwelling people with a mean age of 60 years or older and examined a relationship between any type of PA and a determinant. Two independent reviewers screened 17,277 references and 11 reviews were ultimately included. Of the countries reported, 78% were high-income and zero were lower-income countries. Three of the eleven reviews focused on specific clinical populations living in the community. Only 6% of studies included in all reviews had longitudinal designs. Included studies focused on a variety of types of PA (e.g., total PA, walking, leisure-time PA) and measures of PA, with 76% using self-report, 15% using direct measures (e.g., accelerometry), 3% using both types, and 6% with no outcome measure reported. Physical environment determinants were the most commonly assessed (6/11) with more than 70% of the summarized relationships demonstrating null associations. Three out of four reviews reported a positive relationship between walkability and PA in general community-dwelling older adults. Further prospective longitudinal studies are needed to improve our current understanding of the determinants of PA in community-living older people.

